# Peripheral Precocious Puberty Due to Autonomous Gonadal Activation: A Multicenter Experience

**DOI:** 10.7759/cureus.92316

**Published:** 2025-09-14

**Authors:** Sushil Yewale, Vaman Khadilkar, Nikhil Shah, Shalmi Mehta, Hemchand K Prasad, Chirantap Oza, Aniket Kumbhojkar, Ruma Deshpande, Ankita Maheshwari, Anuradha Khadilkar

**Affiliations:** 1 Department of Growth and Pediatric Endocrinology, Hirabai Cowasji Jehangir Medical Research Institute, Pune, IND; 2 Department of Health Sciences, Savitribai Phule Pune University, Pune, IND; 3 Division of Pediatric Endocrinology, Department of Pediatrics, Surya Children’s Hospital, Mumbai, IND; 4 Department of Pediatric Endocrinology, Endokids Clinic, Ahmedabad, IND; 5 Department of Pediatric Endocrinology, Mehta Hospital, Chennai, IND; 6 Department of Pediatric Endocrinology, EndoGrow Pediatric and Adolescent Endocrine Center, Ahmedabad, IND; 7 Department of Pediatric Endocrinology, Grow Kids Endocrine Clinic, Sangli, IND; 8 Department of Pediatrics, Bharati Vidyapeeth (Deemed to Be University) Medical College, Pune, IND; 9 Department of Pediatrics, Sri Aurobindo Institute of Medical Sciences, Indore, IND

**Keywords:** autonomous gonadal activation, bicalutamide, familial male-limited precocious puberty, familial testotoxicosis, functional ovarian cysts, gonadotropin-independent precocious puberty, letrozole, leuprolide acetate, mccune-albright syndrome, peripheral precocious puberty

## Abstract

Introduction: Peripheral precocious puberty (PP) is far less commonly encountered compared to central precocious puberty (CPP) in pediatric endocrine practice. Long-standing non-diagnosis may cause rapidly advancing bone age, leading to CPP and resultant short stature. We aimed to report the clinical profile and current Indian experience in the management of children with peripheral PP due to autonomous gonadal activation.

Methods: This multicentric retrospective study reports data on 23 children (20 girls) presenting with peripheral PP as a result of autonomous gonadal activation from eight pediatric endocrine centers across India. Their clinicodemographic, anthropometric, and laboratory measurements were reviewed.

Results: The mean ± SD chronological age at the time of presentation was 4.9 ± 2.0 years, and the mean bone age was 7.6 ± 2.6 years. Nine (39%) children were tall for mid-parental height. Thirteen (57%) children were diagnosed with McCune Albright syndrome (MAS), one boy (4%) with familial male-limited PP, and nine (39%) with functional ovarian cysts. Ninety-five percent of girls had menarche as their presenting complaint, with mean age at menarche being 4.6 ± 2.3 years. Ovarian cysts were present in 16 girls, of whom seven (43.8%) had MAS. PP in seven (30%) children progressed to CPP, with mean age of CPP being 6.6 ± 2.1 years. Letrozole was the primary drug of choice, while leuprolide acetate was added in children who progressed to CPP.

Conclusion: We report the clinical profile of children with peripheral PP due to autonomous gonadal activation. Clinical diagnosis and timely intervention to halt the progression of puberty and prevent early epiphyseal fusion, thereby improving final height, along with close follow-up, are vital in the management of these disorders.

## Introduction

Precocious puberty (PP) is defined as the onset of secondary sexual characteristics before the age of eight years in girls and nine years in boys [1,2]. PP may be central or peripheral, that is, gonadotropin-dependent or gonadotropin-independent, respectively. Peripheral PP, or gonadotropin-independent PP (GIPP), is far less common, with its incidence around one-fifth of the total cases of PP [3]. Unlike the classic sequence of secondary sexual development seen in central PP (CPP) (thelarche followed by pubarche followed by menarche in girls, and testicular enlargement followed by pubarche followed by penile enlargement in boys), children with peripheral PP often present with discordant features. Peripheral PP can occur secondary to a multitude of causes, including adrenal pathology such as congenital adrenal hyperplasia (CAH), long-standing undiagnosed or untreated primary hypothyroidism, tumors arising from either the adrenals or gonads, or as a result of autonomous gonadal activation [1,3].

Autonomous gonadal activation encompasses a wide range of disorders, from the more commonly reported functional follicular ovarian cysts to extremely rare conditions like McCune-Albright syndrome (MAS) and familial testotoxicosis, or familial male-limited precocious puberty (FMPP). This heterogeneous group of disorders results in excess secretion of gonadal sex hormones (estrogen or testosterone) in the absence of activation of the hypothalamic-pituitary-gonadal (HPG) axis [1,3,4]. Thus, unlike CPP, which is marked by a surge in gonadotropin-releasing hormone (GnRH) and increased secretion and pulsatility of follicle-stimulating hormone (FSH) and luteinizing hormone (LH) from the pituitary, serum concentrations of these hormones remain suppressed in peripheral PP. Activating mutations, either in the GNAS1 gene in MAS or in the luteinizing hormone/chorionic gonadotropin receptor (LHCGR) gene in FMPP, result in stimulation of the cyclic adenosine monophosphate (cAMP) pathway, thereby causing autonomous gonadal hyperactivity [3,5,6].

Functional follicular ovarian cysts, either isolated or as part of MAS, most commonly present with vaginal bleeding at a very young age in girls, conspicuously absent pubic hair, with or without development of breast tissue (usually Tanner stage II-III), and with or without enhanced growth velocity [7]. Such cases may be wrongly labeled as CPP or as isolated vaginal bleeding due to a foreign body or local trauma if not thoroughly evaluated. Due to the rarity of this condition, a high index of suspicion is required for diagnosis and referral. This is important as long-standing non-diagnosis of such cases leads to recurrent bleeds (causing unfavorable psychological effects) and rapidly progressing bone age leading to CPP and final short stature [1,2,7].

The literature on peripheral PP arising from autonomous activation of gonads is scarce, with very little known about the management of these disorders. There is enough evidence that the use of GnRH agonists (unless there is evidence of CPP) and surgical oophorectomy or cystectomy (as new follicular cysts may develop) are ineffective [7,8]. Broadly, management revolves around blocking sex steroid synthesis and curbing the peripheral actions of these hormones, such as the development of secondary sexual characteristics, menses, and bone age advancement [3,7]. However, there is a paucity of literature in this perspective from the Indian subcontinent. Moreover, early diagnosis and treatment can help prevent recurrent bleeds and slow bone age advancement. Thus, the primary objective of this study was to report the clinical profile and current Indian experience in the management of children with peripheral PP due to autonomous gonadal activation.

## Materials and methods

Subjects

This multicenter retrospective study reports data on 20 girls among 23 children presenting with peripheral PP as a result of autonomous gonadal activation from eight pediatric endocrine centers (eight from Jehangir Hospital, Pune, and 15 from collaborating centers) across India between September 2010 and October 2024. The study was approved by the Institutional Ethics Committee. Written informed consent was obtained from the parents for the reporting of deidentified data. Children with a clinical and laboratory diagnosis of MAS, FMPP, or autonomous functional follicular ovarian cyst were included. Those with central precocious puberty (CPP), as well as children with other causes of peripheral PP such as congenital adrenal hyperplasia, adrenal or gonadal tumors, primary hypothyroidism, or inadvertent exposure to exogenous sex steroids, were excluded from the study.

Demography, clinical history, and anthropometry

Demographic data as well as anthropometric measurements were obtained for each subject from existing medical records. Clinical history and examination findings, including age at presentation, history of fractures, presence or absence of café-au-lait pigmentation, family history, evolution into CPP, presence or absence of other endocrinopathies, and treatment details, were recorded using standardized proformas. Height, weight, and body mass index (BMI) were converted into Z-scores using ethnic-specific reference data [9]. Mid-parental height (MPH), or target height, was calculated by taking the mean of both parents’ heights and adding or subtracting 6.5 cm for boys and girls, respectively [10]. Height Z-scores were calculated for both parents individually, and MPH Z-scores were calculated by taking the mean of both parents’ height Z-scores [11]. Tall stature for MPH was defined as a difference between the height Z-score and the MPH Z-score greater than +1.5 SD [12]. Sexual maturity rating (SMR) was performed by Tanner staging, which comprises five stages for breast, pubic hair, and male genital development, and three stages for axillary hair development [1]. Breast budding in girls and an increase in testicular volume of ≥4 mL are considered the first signs of pubertal onset. When the normal sequence of thelarche, pubarche, and menarche in girls, and testicular enlargement, pubarche, and genital enlargement in boys is maintained (as in CPP), PP is said to be "concordant," whereas in the "discordant" type of PP (as in peripheral PP), there is disruption of the normal concordance, for example, penile enlargement and advanced pubic hair stage with prepubertal testes or menarche with early stages of thelarche [1,2].

Biochemical assessments

Biochemical data, including basal and stimulated concentrations (if available) of serum LH, FSH, and estradiol or testosterone, were obtained. Estradiol levels greater than 10 pg/mL and testosterone levels greater than 25 ng/dL were considered within pubertal ranges [2]. A serum LH concentration of ≥0.3 mIU/L (along with other supportive anthropometric and radiologic evidence), suggestive of CPP, was used to exclude such cases [1,2]. Additionally, serum concentrations of thyroid-stimulating hormone (TSH), prolactin, alpha-fetoprotein (AFP), and beta-hCG were also recorded. Clinical exome sequencing was performed in only three children with MAS, details of which were recorded.

Radiological assessments

Bone age was determined by the Tanner-Whitehouse 3 method by assessing the radiograph of the left hand and wrist [13,14]. The same radiograph was also reviewed for the presence of any cysts, typically seen in MAS, in addition to radiographs of other sites, wherever indicated. The size and morphology of the uterus and ovaries were assessed using transabdominal ultrasound of the pelvis. Uterine length ≥35 mm and/or uterine volume >2 mL, with transformation from a tubular to a pear-shaped appearance, were considered radiological markers of pubertal onset [1,2,15]. Details of 99mTc-MDP (technetium-99m methylene diphosphonate) scintigraphy scans (for fibrous dysplasia), if performed, were recorded.

Diagnosis

The diagnosis of peripheral PP was based on elevated basal concentrations of estradiol (>10 pg/mL) or testosterone (>25 ng/dL) in conjunction with suppressed basal concentrations of LH and FSH (<0.3 mIU/L) in a child with clinical signs of puberty [3]. The diagnosis of MAS was made on clinical grounds when two of the three classic features were present with peripheral PP (fibrous dysplasia of bone, café-au-lait skin pigmentation, and/or hyperfunctioning endocrinopathies). Peripheral PP with autonomous ovarian cysts on pelvic ultrasound, in the absence of any other features of MAS, was characteristic of autonomous functional follicular ovarian cysts [16]. FMPP was diagnosed based on history and presentation with peripheral PP after other causes such as MAS, CAH, hypothyroidism, and tumors were ruled out [3,6]. Genetic testing for most cases was not performed because of poor diagnostic yield as a result of postzygotic somatic mutations that lead to mosaic tissue distribution [17].

Statistical analysis

All statistical analyses were carried out using the IBM SPSS Statistics for Windows, Version 26 (Released 2019; IBM Corp., Armonk, New York). Normality testing was performed for all variables before conducting any statistical analyses. Cases were categorized as autonomous functional follicular ovarian cysts, MAS, or FMPP. Frequency distribution was used to study the salient features seen in each disorder.

## Results

The mean ± SD chronological age of the subjects at presentation was 4.9 ± 2.0 years, while the mean bone age was 7.6 ± 2.6 years. Height-for-age Z-scores and MPH Z-scores averaged 0.32 ± 1.32 and −0.91 ± 0.75, respectively, with nine (39%) children being tall for MPH (+1.5 SD above MPH Z-score) at presentation.

**Figure 1:**
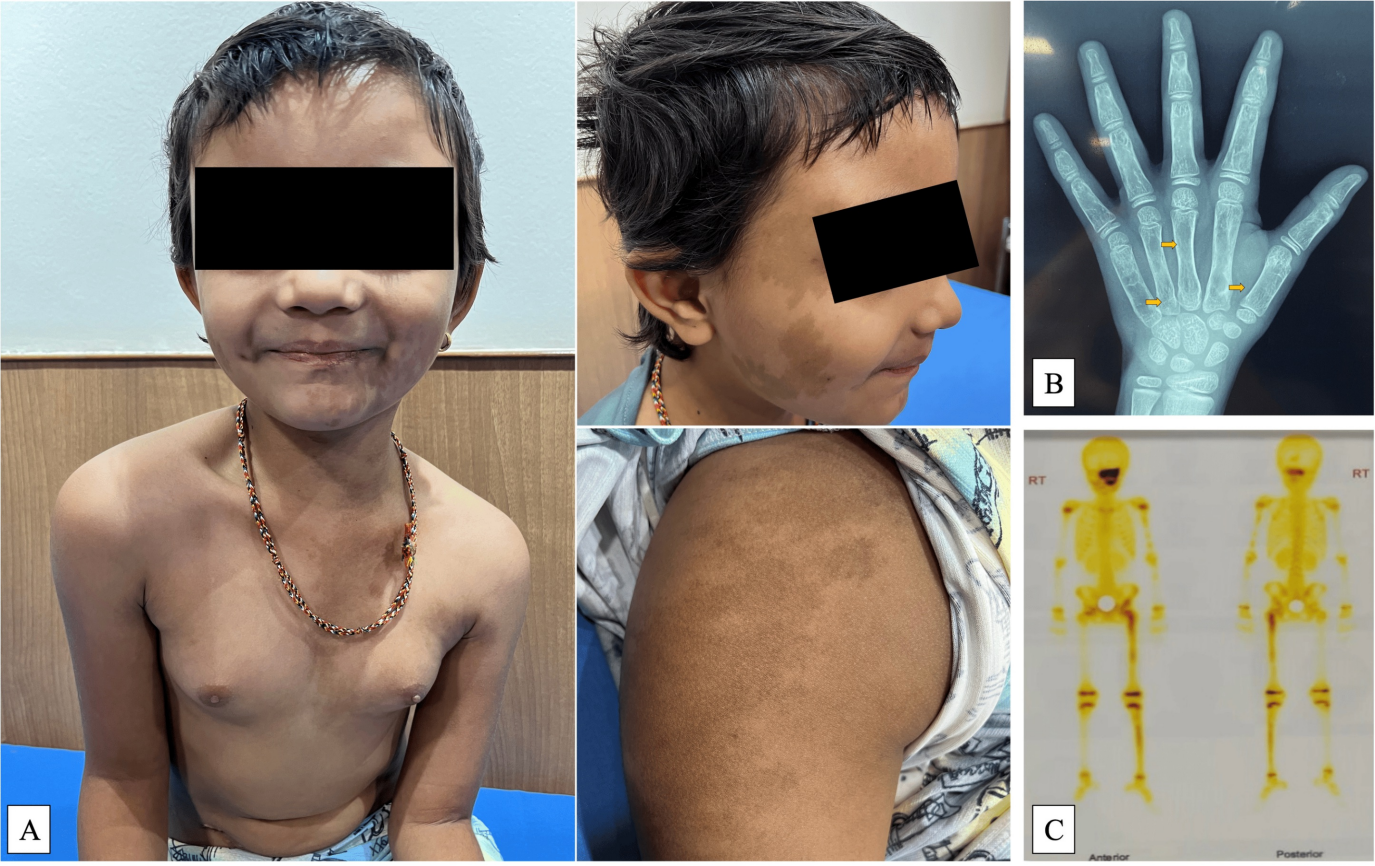
6.6-year-old girl with McCune-Albright syndrome. A. Multiple café-au-lait macules with irregular borders and bilateral breast enlargement, stage 3. B. Polyostotic fibrous dysplasia with left-hand bone age radiograph showing multiple cystic lesions. C. Increased tracer uptake seen on the left side of the skull on bone scan.

**Figure 2:**
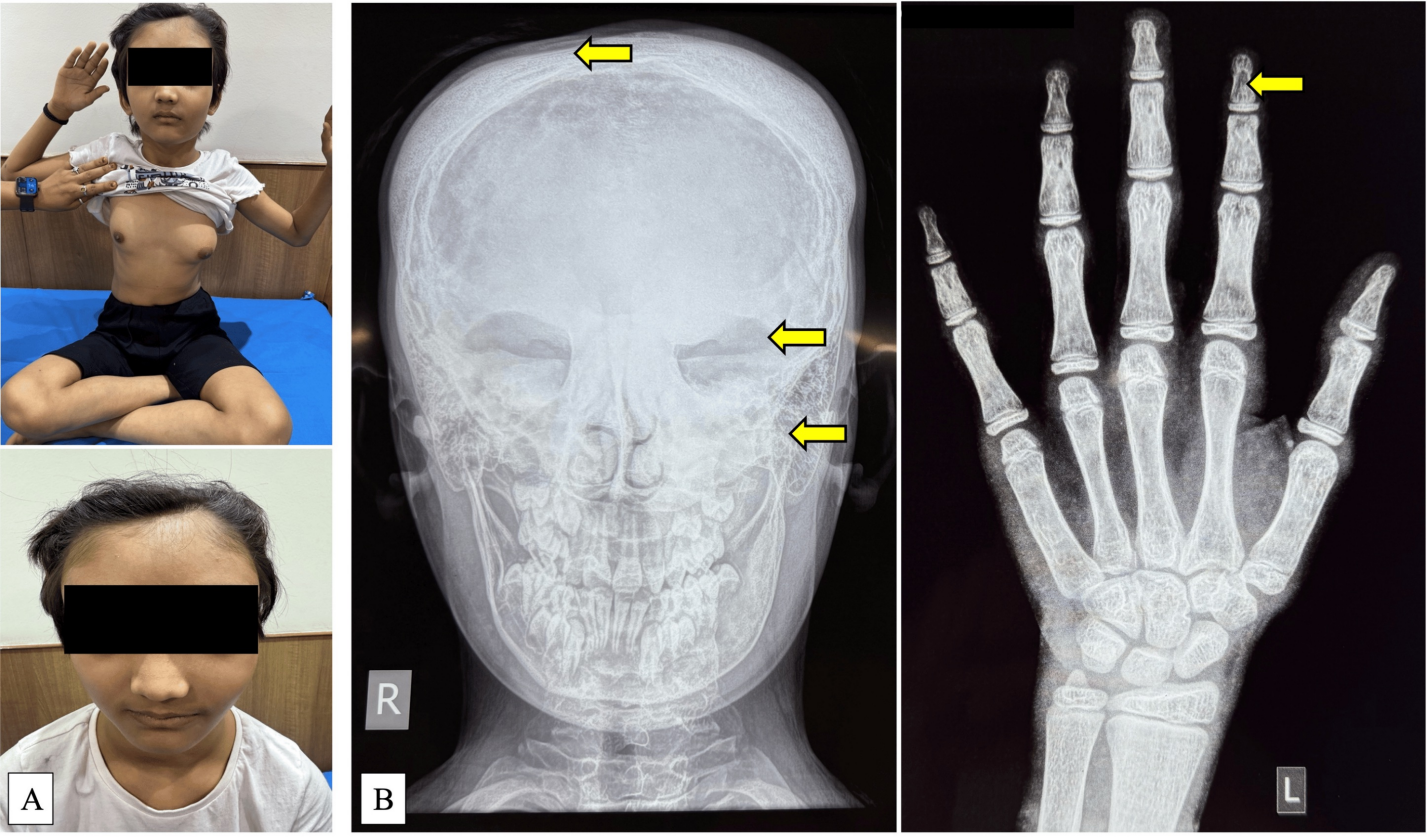
8.3-year-old girl with McCune-Albright syndrome. A. Bilateral breast development, stage 3/4, and facial asymmetry (craniofacial fibrous dysplasia). B. Skull radiograph showing craniofacial fibrous dysplasia and multiple cystic lesions on left-hand bone age radiograph.

**Figure 3:**
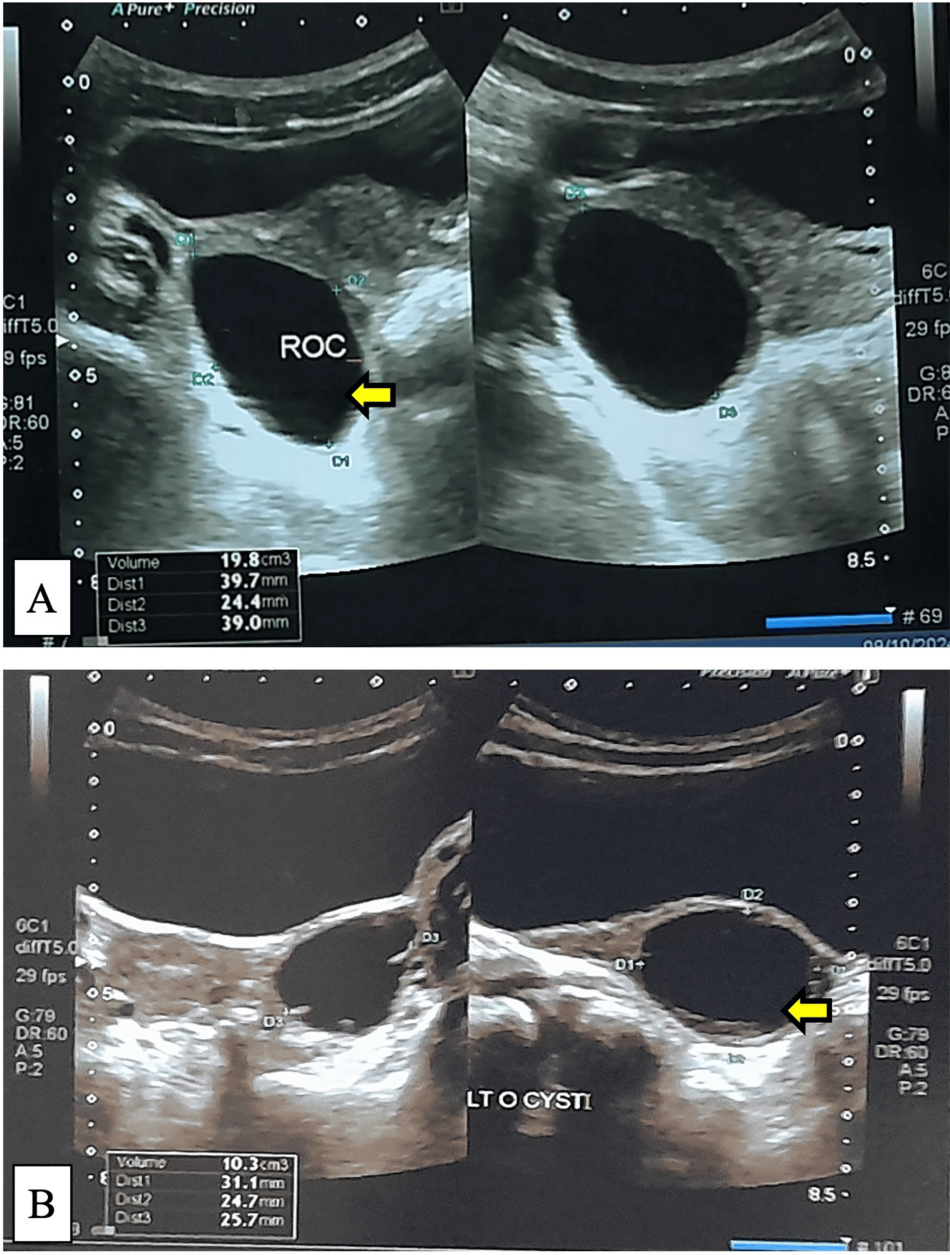
Ovarian cyst on pelvic ultrasound. A. 5.7-year-old girl presenting with breast budding; pelvic ultrasound shows a right ovarian cyst measuring 3.9 × 2.4 × 3.9 cm. B. 4.6-year-old girl presenting with stage 2 breast enlargement and menarche; pelvic ultrasound shows a left ovarian cyst measuring 3.1 × 2.6 × 2.5 cm.

Among our cohort of 23 children, 13 (57%) were diagnosed with MAS, one boy (4%) with FMPP, and the remaining nine (39%) with autonomous functional follicular ovarian cysts. Tables 1, 2 illustrate the characteristic features of each disorder. Figure 1A and Figure 2A depict clinical signs of MAS in two of our patients. Ninety-five percent of girls presented with menarche, with a mean age at menarche being 4.6 ± 2.3 years. By contrast, in the two boys with MAS and the one with FMPP, the first symptom was café-au-lait skin pigmentation and increased phallic/testicular size, respectively. Ovarian cysts were present in 16 girls, of whom seven (43.8%) had MAS (Figure 3). 

**Table 1:** Clinicodemographic, biochemical, and radiological characteristics of children with peripheral precocious puberty due to MAS, functional ovarian cysts, and FMPP Data presented as mean(SD), median (25th centile, 75th centile) and n (%). MPH, mid-parental height; BA, bone age; CA, chronological age; LH, luteinizing hormone; FSH, follicle-stimulating hormone; MAS, McCune Albright syndrome; FMPP, familial male-limited precocious puberty.

Variables	McCune-Albright syndrome (n=13)	Functional ovarian cysts (n=9)	Familial male-limited precocious puberty (n=1)
Gender			
Males	2 (15.4%)	-	1 (100%)
Females	11 (84.6%)	9 (100%)	-
Age at presentation (years)	4.6 (2.2)	5.3 (1.7)	7.5
Height Z-score	0.02 (1.6)	0.6 (0.8)	1.3
MPH Z-score	-1.1 (0.8)	-0.7 (0.6)	-0.2
Bone age (years)	7.5 (3.2)	7.2 (1.7)	9.1
BA/CA ratio	1.4 (0.3)	1.3 (0.3)	1.2
LH (mIU/L)	<0.07	<0.07	<0.12
FSH (mIU/L)	<0.3	<0.3	<0.11
Estradiol (pg/mL)	60.5 (28.1, 223.9)	56.0 (28.5, 360)	NA
Testosterone (ng/dL)	97.3 (134.8)	NA	488
Size of uterus (length in cm)	4.3 (1.0)	4.5 (1.1)	NA
Ovarian volume (in mL)	1.5 (0.7, 8.0)	1.4 (0.9, 4.2)	NA
Ovarian cysts	7 (63.6%)	9 (100%)	NA
Size of cyst (largest dimension in cm)	2.5 (1.8)	4.0 (0.8)	NA
Treatment of choice	Letrozole 2.5 mg/day (± spironolactone 25 mg/day and flutamide 125 mg/day)	Letrozole 2.5 mg/day	Anastrazole 1 mg/day + Bicalutamide 50 mg/day

**Table 2:** Clinical profile of children with peripheral precocious puberty HAZ, height-for-age z-score; SMR, sexual maturity rating; A, axillary hair staging; P, pubic hair staging; G, genital staging; B, breast staging (left, right); T, testicular volume in mL (left, right).

Case no.	Sex	Age at presentation (in years)	Age at menarche (in years)	HAZ	MPHZ	Bone age (in years)	SMR	Cafe-au-lait	Fractures	Bone cysts	Ovarian cysts	CPP	Other endocrinopathies
McCune Albright syndrome													
1.	F	3.2	1.0	2.01	-1.28	4.0	A1 P1 B2 B2 M1	+	Carpals	-	+	+ (4.6 years)	-
2.	F	2.7	-	-2.08	-1.71	3.8	A1 P1 B3 B3 M0	+	B/L femur, left tibia, and fibula	+	+	-	Hyperthyroidism
3.	F	7.0	7.0	-0.42	-1.93	14.0	A3 P3 B4 B4 M1	+	Left femur	+	+	-	-
4.	F	6.6	2.0	-0.36	-1.38	8.2	A1 P1 B3 B3 M1	+	Left humerus, B/L femur	+	+	-	-
5.	M	1.7	NA	-1.86	-0.21	-	A1 P1 G2 T2 T2	+	-	-	NA	-	-
6.	F	4.1	4.1	-1.02	-1.34	-	A1 P1 B1 B1 M1	-	-	+	-	+ (6.3 years)	-
7.	F	3.2	3.2	1.54	0.19	5.0	A1 P1 B1 B1 M1	+	-	-	+	+ (8 years)	-
8.	F	5.5	5.5	-0.64	-0.89	6.0	A1 P1 B2 B2 M1	-	-	+ (Femur)	+	-	-
9.	F	0.8	4.7	1.09	0.16	7.0	A2 P3 B2 B2 M1	+	-	+ (B/L metacarpals)	+	+ (3.8 years)	Hyperthyroidism
10.	F	4.5	0.5	-2.36	-2.22	9.0	A1 P1 B3 B4 M1	+	Right humerus, Right femur	+ (Right humerus, Right femur)	-	-	Hyperthyroidism, hypophosphatemic rickets
11.	F	6.0	6.0	1.80	-	9.8	A2 P2 B3 B3 M1	-	-	+ (left 2^nd^metacarpal)	-	-	-
12.	M	6.0	NA	1.82	-	9.0	A1 P3 G5 T8 T25	+	Left femur	+ (left femur)	NA	-	-
13.	F	8.3	8.9	0.80	-1.20	11.1	A1 P3 B4 B3 M1	+	-	+ (distal phalanges)	-	-	-
Functional ovarian cysts													
14.	F	4.6	4.6	1.02	-0.55	5.7	A1 P1 B2 B2 M1	-	-	-	+	-	-
15.	F	4.4	3.2	1.69	-0.94	6.1	A1 P3 B3 B3 M1	-	-	-	+	-	-
16.	F	6.8	6.8	2.17	-	10.2	A1 P2 B2 B2 M1	-	-	-	+	-	-
17.	F	2.2	2.1	-0.03	0.32	-	A1 P1 B1 B1 M1	-	-	-	+	+	-
18.	F	6.5	6.5	0.14	-0.68	6.8	A1 P1 B2 B2 M1	-	-	-	+	+ (8.9 years)	-
19.	F	7.8	7.8	0.66	-	-	A2 P2 B2 B2 M1	-	-	-	+	+ (8.1 years)	-
20.	F	4.7	4.7	0.06	-1.63	5.6	A1 P1 B2 B2 M1	-	-	-	+	-	-
21.	F	4.6	4.6	0.35	-	8.1	A1 P2 B2 B2 M1	-	-	-	+	-	-
22.	F	5.7	-	-0.36	-0.80	5.5	A1 P1 B2 B2 M0	-	-	-	+	-	-
Familial male-limited precocious puberty													
23.	M	7.5	NA	1.27	-0.20	9.1	A2 P3 G4 T3 T3	-	-	-	NA	-	-

Nine (69.2%) children with MAS had a history of fractures, with a mean age at fracture being 6.1 ± 4.1 years. Bone cysts were reported in 10 (76.9%) children (Figure 1B, C, and Figure 2B). Three children had hyperthyroidism, and one girl had hypophosphatemic rickets as part of the other hyperfunctioning endocrinopathies seen in MAS. Clinical exome sequencing was performed in three children with MAS (for research purposes); one girl was found to have an activating mutation in the GNAS gene (c.60>T/p.Arg201Cys) in a peripheral blood specimen, while the remaining two (one skin biopsy) did not yield any mutation.

PP in seven (30%) children progressed to CPP later (2.4 ± 1.5 years after diagnosis), with a mean age at CPP being 6.6 ± 2.1 years. Letrozole (aromatase inhibitor) at a dose of 2.5 mg/day was the primary drug of choice for treating peripheral PP in 95% of cases, while depot leuprolide acetate (GnRHa analogue, 11.25 mg intramuscularly every three months, 140-300 mg/kg/month) was added in all seven children who progressed to CPP. One boy with FMPP was treated with anastrozole (aromatase inhibitor, 1 mg/day) and bicalutamide (nonsteroidal antiandrogen, 50 mg/day), while spironolactone (25 mg/day) and flutamide (125 mg/day) were used as antiandrogens in addition to letrozole in a boy with MAS. All three children with hyperthyroidism had undetectable TSH concentrations with raised free T3 and free T4 and were treated with carbimazole at a dose of 0.25-0.5 mg/kg/day.

## Discussion

Our multicenter study highlights the clinical heterogeneity seen in children with disorders of autonomous gonadal activation from multiple pediatric endocrine centers across India. Our cohort of children with these extremely rare disorders, viz. MAS, FMPP, or functional ovarian cysts, all presented with features of peripheral PP during early childhood, with their mean bone age significantly advanced compared to their chronological age, thus resulting in much higher height-for-age Z-scores. Menarche was the most common presenting feature due to the discordant nature of PP, with a mean age at menarche much below the accepted norm and a cause of distress to the child as well as the parents. Among those with MAS, apart from PP, fibrous dysplasia of bone was the most commonly reported feature, followed by café-au-lait skin pigmentation and hyperthyroidism. Nearly one-third of children with peripheral PP progressed to CPP at a later stage, emphasizing the need for close monitoring and follow-up, as addition of a GnRH analogue may be warranted in such cases.

The mean age of presentation in our cohort was similar to that reported in a study from China on children with PP and growth hormone (GH) excess as part of MAS [18]. The median advancement of bone age over chronological age was also comparable to their study, whereas that reported in a case series on peripheral PP due to ovarian causes from southern India was much lower [8,18]. This difference could be attributed to a shorter duration of symptoms and a smaller sample size (four girls), which may not be entirely representative of the condition [8]. The typical female preponderance in our study (20 girls and only three boys) is consistent with prior reports from Italy and a European collaborative project [19,20]. This may be due to the ability of both ovarian theca and granulosa cells to produce steroids versus only Leydig cells in the testes (and not Sertoli cells), as suggested by Rey et al. [21].

Hypophosphatemic rickets was seen in one girl with MAS in our cohort. Although often considered a separate feature among hyperfunctioning endocrinopathies seen in MAS, hypophosphatemia is in truth a complication of fibrous dysplasia (producing FGF23), and an important predictor of future fracture risk [5]. The broad clinical spectrum of MAS reported from our study is an illustration of Happle’s hypothesis of mosaicism (secondary to post-zygotic mutation in the *GNAS1* gene) in this disorder [17]. As a result, gene mutation analysis had a poor diagnostic yield in our study, highlighting the role of clinical diagnosis. Similarly, Lumbroso et al. in their systematic search for gene mutations in 113 patients with MAS found a mutation in peripheral blood in only 46% of individuals where the classic triad was present, while the yield dropped to 21% and 8% in subjects with two classic signs and one sign, respectively [19]. The odds of a genetic diagnosis may improve if performed on target affected tissues such as bone; however, diagnostic biopsies have to be processed as fresh or fresh-frozen material, and false negatives may occur if the biopsy specimen comprises normal tissue. Moreover, the odds of identifying a positive mutation in skin biopsies (as affected tissue) are significantly lower owing to the mosaic distribution of affected cells [5]. Testotoxicosis or FMPP, generally considered a diagnosis of exclusion, should be suspected in boys with peripheral PP and a strong family history, due to its autosomal dominant inheritance. However, it could also present sporadically, as seen in our case, wherein all other causes of peripheral PP, including MAS, CAH, primary hypothyroidism, and tumors, were ruled out before arriving at a diagnosis. Similar cases with de novo mutations have been reported in the past [6,22].

Several different pharmacological strategies have been tried in the past to halt pubertal progression with either monotherapy or combined therapy with agents such as letrozole, medroxyprogesterone, and tamoxifen, and have shown variable outcomes [23,24]. The predominant use of letrozole in girls from our cohort was due to its proven role not only in cessation of vaginal bleeding but also in improvement of final height outcomes, as evidenced by Estrada et al. in their study on 28 girls with MAS and PP [25]. The aim of treatment in males with autonomous gonadal activation is twofold: block androgen synthesis/action (controlling androgenization) as well as estrogen synthesis (preventing epiphyseal fusion) [7,26,27]. This was achieved with the use of competitive androgen receptor blocker bicalutamide and third-generation aromatase inhibitor anastrazole in our subjects, similar to that reported by Tessaris et al. [28]. None of the patients from our cohort required surgical intervention in the form of either oophorectomy or orchidectomy. A case series from India previously highlighted the importance of conservative management in functional ovarian cysts, as there is a high risk of recurrence even after surgical removal, thus limiting its role only in cases with a high risk of ovarian torsion or pharmacologic treatment failure [8].

Progression to CPP is not uncommon in children with peripheral PP, particularly when bone age advancement is beyond 11 years [7]. Treatment with GnRH analogue was the mainstay in such children from our cohort, as also suggested previously in several studies [18,25,29]. Additionally, in children with MAS, the trajectory of growth may be impacted by the presence of other endocrinopathies, such as accelerated growth in hyperthyroidism and GH excess, and growth deceleration in hypophosphatemia or Cushing syndrome, thus necessitating vigilant and close follow-up [18,25,30].

The strengths of our study were its multicentric design and comprehensive clinical phenotyping of disorders of autonomous gonadal activation. Moreover, the sample size was considered to be fairly moderate given the rarity of this group of disorders. Lack of longitudinal data on final adult height outcomes of these children after treatment was a major limitation of the study. Lack of a genetic diagnosis (though not always warranted, particularly in MAS, due to poor diagnostic yield as a result of post-zygotic somatic mutations) and intercenter variability in laboratory assays, along with lack of data on recurring bone age or biochemical assessments and reliance on descriptive rather than comparative statistics, were among other limitations.

## Conclusions

In conclusion, we report the diverse clinical profile and Indian experience in the management of children with peripheral PP due to autonomous gonadal activation. Clinical diagnosis and timely intervention to halt the progression of puberty and prevent early epiphyseal fusion, thereby improving final height, along with close monitoring and follow-up, are vital in the management of these disorders.
